# Clinical characteristics, management, and prognosis of ipilimumab-induced hypophysitis: a retrospective analysis of 76 cases

**DOI:** 10.3389/fendo.2026.1828090

**Published:** 2026-06-30

**Authors:** Ling Wu, Yuping Yang, Qiongli Su

**Affiliations:** Department of Pharmacy, Zhuzhou Hospital Affiliated to Xiangya School of Medicine, Central South University, Zhuzhou, Hunan, China

**Keywords:** adrenal insufficiency, CTLA-4 inhibitor, endocrine toxicity, hypophysitis, ipilimumab, pituitary dysfunction

## Abstract

**Background:**

Ipilimumab, a cytotoxic T-lymphocyte associated antigen-4 inhibitor, is associated with immune-related endocrine adverse events, among which hypophysitis represents a distinctive but incompletely characterized toxicity. This study aimed to systematically summarize the clinical characteristics, therapeutic approaches, and prognostic outcomes of ipilimumab-induced hypophysitis.

**Methods:**

We conducted a retrospective, case-based synthesis of ipilimumab-induced hypophysitis. PubMed, EMBASE, Web of Science, WanFang Data, and CNKI were systematically searched from database inception to February 1, 2026, to identify eligible case reports and case series using combinations of terms related to ipilimumab, hypophysitis, and immune-related adverse events. The methodological quality of included reports was assessed using the Joanna Briggs Institute Critical Appraisal Checklist.

**Results:**

A total of 76 patients were included. The median age was 58 years (range 26–82), and 61.8% were male. The median time to onset was 9 weeks (range 1.6–37), with more than half occurring within the first 10 weeks of treatment. Headache (68.0%) and fatigue (64.0%) were the most common symptoms. Secondary adrenal insufficiency predominated, with low cortisol and ACTH levels detected in 93.4% and 89.8% of evaluated patients, respectively. Central hypothyroidism and hypogonadism were also frequent. Pituitary enlargement was observed in 71.0% of patients undergoing magnetic resonance imaging. Glucocorticoid replacement constituted the primary treatment. Clinical improvement occurred in 82.9% of patients; however, persistent endocrine dysfunction remained common, and complete hormonal recovery was rare (7.9%). Ipilimumab rechallenge was uncommon. According to the WHO–UMC system, most cases (80.3%) were classified as probable.

**Conclusion:**

Ipilimumab-induced hypophysitis typically occurs early in treatment and is characterized by secondary adrenal insufficiency with frequent pituitary enlargement. Although symptoms often improve with hormone replacement, recovery of pituitary function is uncommon, warranting long-term endocrine monitoring.

## Introduction

Immune checkpoint inhibitors (ICIs) have fundamentally reshaped the management of advanced malignancies by restoring antitumor immune responses through blockade of inhibitory immune pathways (1). Among these agents, ipilimumab, a fully human monoclonal antibody targeting cytotoxic T-lymphocyte–associated antigen-4 (CTLA-4), was the first immune checkpoint inhibitor approved for advanced melanoma and has demonstrated durable survival benefits across multiple tumor types (2). By inhibiting CTLA-4–mediated downregulation of T-cell activation, ipilimumab enhances cytotoxic immune responses against tumor cells (3). However, this immune activation is not tumor-specific and may disrupt self-tolerance, leading to immune-related adverse events (irAEs) affecting multiple organ systems (4).

Endocrine toxicities are among the most distinctive and clinically significant irAEs associated with CTLA-4 blockade. Hypophysitis represents a characteristic complication of ipilimumab therapy and occurs at a substantially higher frequency than with PD-1 or PD-L1 inhibitors (5). The condition is believed to result from immune-mediated inflammation of the pituitary gland, potentially involving CTLA-4 expression on pituitary cells and antibody-dependent cytotoxic mechanisms (6). Clinically, ipilimumab-induced hypophysitis often presents with nonspecific manifestations such as headache, fatigue, nausea, dizziness, and visual disturbances, which may delay recognition (7). Biochemically, secondary adrenal insufficiency is the most frequent hormonal abnormality, frequently accompanied by central hypothyroidism and hypogonadism. In rare cases, involvement of the posterior pituitary may lead to diabetes insipidus (8). Neuroimaging plays a central role in diagnosis. Magnetic resonance imaging typically demonstrates diffuse pituitary enlargement, homogeneous or heterogeneous contrast enhancement, and, occasionally, thickening of the infundibulum (9). However, imaging findings are not uniform and may vary depending on the timing of assessment. In some instances, metabolic imaging has detected pituitary inflammation before overt clinical or biochemical abnormalities emerge, underscoring the dynamic nature of this immune-mediated process (10). Although numerous case reports and small case series have described ipilimumab-associated hypophysitis, important questions remain regarding its demographic patterns, temporal onset, spectrum of endocrine dysfunction, management strategies, and long-term outcomes (11). Persistent hormonal deficiency appears common, yet reported recovery rates vary considerably, and the clinical predictors of recovery remain unclear (12). As the use of ICIs continues to expand across malignancies and combination regimens become increasingly common, a comprehensive synthesis of accumulated clinical evidence is essential to optimize early recognition and long-term management (13).

In this context, we conducted a retrospective, case-based analysis of 76 published cases of ipilimumab-induced hypophysitis to systematically characterize patient demographics, time to onset, clinical presentation, laboratory and imaging features, therapeutic interventions, and prognostic outcomes. By delineating the typical phenotype and long-term endocrine sequelae of this distinctive toxicity, we aim to provide clinically relevant insights to guide monitoring and individualized endocrine management during CTLA-4–targeted therapy.

## Methods

### Study design and search strategy

This study was designed as a retrospective, case-based synthesis of published reports describing ipilimumab-induced hypophysitis. The objective was to systematically summarize the clinical characteristics, management strategies, and prognostic outcomes of this immune-related endocrine adverse event. PubMed, EMBASE, Web of Science, WanFang Data, and CNKI were systematically searched from database inception to February 1, 2026, to identify eligible case reports and case series. The search strategy combined Medical Subject Headings (MeSH) and free-text keywords related to ipilimumab, hypophysitis, and immune-related adverse events. The core search terms included: (“ipilimumab” OR “CTLA-4 inhibitor” OR “cytotoxic T-lymphocyte–associated antigen-4 inhibitor” OR “immune checkpoint inhibitor” OR “ICI”) AND (“hypophysitis” OR “pituitary inflammation” OR “hypopituitarism”) AND (“immune-related adverse event” OR “irAE” OR “endocrinopathy”). No restrictions were applied regarding patient age, sex, ethnicity, or geographic region. Reference lists of eligible articles were manually screened to identify additional relevant studies.

### Inclusion and exclusion criteria

Eligible publications included case reports or case series describing patients who developed hypophysitis during or following ipilimumab therapy, with sufficient clinical, biochemical, or imaging evidence to support the diagnosis. Studies were included regardless of patient age, sex, or geographic origin. Articles were excluded if they were review papers, mechanistic or experimental studies, conference abstracts without individual patient data, animal studies, or reports lacking adequate clinical information to confirm the diagnosis. When overlapping cases were identified across publications, the most comprehensive and detailed report was retained for analysis.

### Study selection and data extraction

Two investigators independently screened titles, abstracts, and full texts for eligibility. Discrepancies were resolved through discussion and consensus. For each eligible case, the following data were systematically extracted: demographic characteristics (age, sex, country), underlying malignancy, ipilimumab dosage and treatment regimen, time to symptom onset, clinical manifestations, laboratory parameters (including cortisol, ACTH, TSH, free thyroxine, gonadotropins, prolactin, and sodium levels), pituitary imaging findings, therapeutic interventions (glucocorticoids and other hormone replacement), ipilimumab management (continuation, interruption, or discontinuation), clinical outcomes, hormonal recovery status, and rechallenge information.

### Quality assessment of case reports

The methodological quality of included case reports and case series was evaluated using the Joanna Briggs Institute (JBI) Critical Appraisal Checklist for Case Reports. Each domain was assessed independently by two reviewers, and disagreements were resolved by consensus.

### Causality assessment

Causality between ipilimumab exposure and the development of hypophysitis was assessed using the World Health Organization–Uppsala Monitoring Centre (WHO–UMC) criteria. Cases were classified as “certain,” “probable,” or “possible” according to the temporal sequence of drug administration, clinical response after discontinuation, and the likelihood of alternative etiologies.

### Statistical analysis

Descriptive statistics were performed using SPSS version 24.0. Continuous variables were summarized as medians with ranges, while categorical variables were presented as frequencies and percentages.

## Results

### Study selection

**Figure 1:**
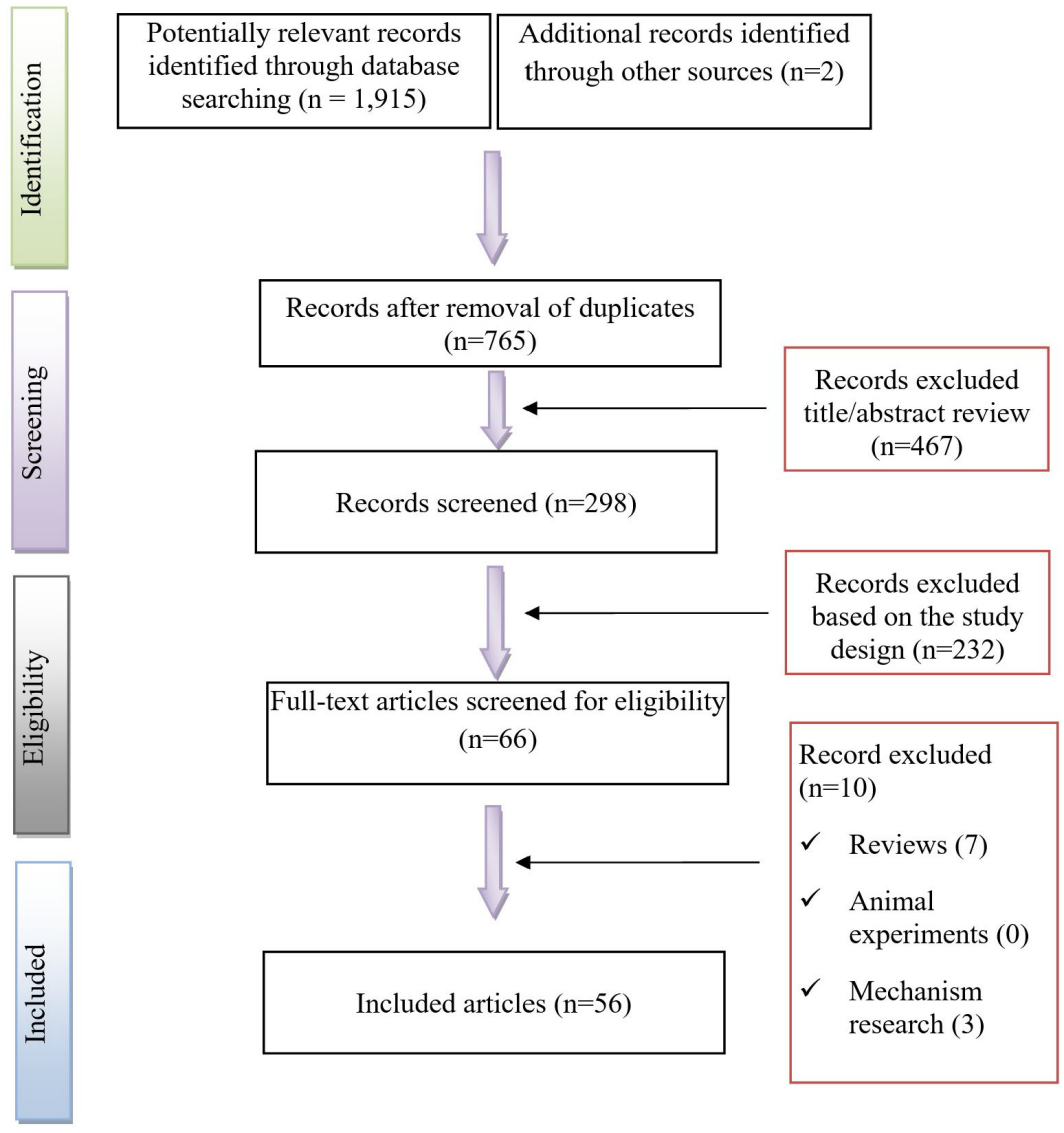
Flowchart illustrating the study selection process for inclusion.

A total of 1,915 records were identified through database searches, with an additional two records obtained from other sources. After removal of duplicates, 765 records remained for screening (**Figure 1**). Following title and abstract review, 467 records were excluded. The full texts of 66 articles were subsequently assessed for eligibility, of which 17 were excluded (7 reviews and 3 mechanistic studies). Ultimately, 56 eligible articles were included in the final analysis (6, 14–68) These reports described 76 patients with ipilimumab-induced hypophysitis, whose baseline characteristics are summarized in **Supplementary Table 1**. Overall, the methodological quality of the included reports was acceptable across the eight domains of the Joanna Briggs Institute Critical Appraisal Checklist. Most cases clearly documented demographic data, clinical presentation, endocrine and imaging findings, therapeutic interventions, and outcomes. Detailed item-level quality assessments are provided in **Supplementary Table 2**.

### Basic characteristics

As shown in **Table 1**, the overall cohort included 76 patients with ipilimumab-induced hypophysitis. The median age was 58 years (range, 26–82), and 47 patients were male (61.8%). Cases were reported from multiple countries, most frequently the United States (40.8%), followed by Germany (10.5%), the United Kingdom (6.6%), Australia (6.6%), and Belgium (6.6%). The most common underlying malignancy was melanoma (73.7%), followed by renal cell carcinoma (10.5%) and prostate cancer (5.3%). Symptom onset data were available for 73 patients, with a median time to onset of 9 weeks (range, 1.6–37) after initiation of ipilimumab. More than half of the cases occurred within the first 10 weeks of treatment (56.2%), followed by 11–20 weeks in 27.4% of cases, indicating a predominantly early-onset pattern. Among patients with reported dosage information, 3 mg/kg was the most frequently used regimen (64.3%), followed by 1 mg/kg and 10 mg/kg. Regarding baseline treatment exposure, 49 patients received ipilimumab without reported concomitant or sequential PD-1/PD-L1 inhibitor therapy. Concomitant medications were documented in 27 patients, most commonly nivolumab, gemcitabine, and cisplatin.

**Table 1:** Baseline profiles of 76 patients with ipilimumab-induced hypophysitis.

Parameter	Classification	Value
Gender (76)^a^	Male	47 (61.8%)
	Female	29 (38.2%)
Age (74)^a^	Years	58 (26, 82)b
Country (76)^a^	USA	31 (40.8%)
	Germany	8 (10.5%)
	UK	5 (6.6%)
	Australia	5 (6.6%)
	Belgium	5 (6.6%)
	France	3 (3.9%)
	Japan	3 (3.9%)
	Netherlands	3 (3.9%)
	Norway	3 (3.9%)
	Switzerland	2 (2.6%)
	China, Canada , Czech Republic, Iceland, Ireland, Italy, Spain, Portugal	8 (10.5%)
Symptom onset time (73)^a^	Weeks	9 (1.6, 37)b
	1–10	41 (56.2%)
	11–20	20 (27.4%)
	21–30	4 (5.5%)
	31–37	8 (11.0%)
Indication (76)^a^	Melanoma	56 (73.7%)
	Renal cell carcinoma	8 (10.5%)
	Prostate cancer	4 (5.3%)
	Lung cancer	2 (2.6%)
	Other cancers	6 (7.9%)
Disease history (19)^a^	Hypertension, type 2 diabetes mellitus, hyperlipidemia, COPD, pulmonary embolism, atrial fibrillation, congestive heart failure, hypothyroidism, osteoporosis, cerebral aneurysm, hashimoto thyroiditis	19 (25.0%)
Dosage (42)^a^	1 mg/kg	7 (16.7%)
	3 mg/kg	27 (64.3%)
	10 mg/kg	7 (16.7%)
	Other dosage	1 (2.4%)
Concomitant medications (27)^a^	Nivolumab, gemcitabine, cisplatin	27 (35.5%)

^a^ Represents the number of patients with this parameter out of 76 patients; ^b^Median (minimum, maximum).

### Clinical manifestations

The most common presenting symptoms of the 76 patients were headache (68.0%) and fatigue or malaise (64.0%), reflecting the frequent involvement of the pituitary gland and secondary adrenal insufficiency. Gastrointestinal manifestations, including nausea, vomiting, or anorexia, were reported in 28.0% of cases. Electrolyte abnormalities were less common but clinically relevant; hyponatremia was documented in 12.0% of patients. Visual disturbances, including blurred vision or visual field impairment, were observed in 10.7%, likely related to pituitary enlargement and mass effect. Dizziness or vertigo occurred in 6.7%, while polyuria or polydipsia suggestive of posterior pituitary involvement was noted in 5.3%. Other less frequent manifestations included fever (4.0%) and a range of nonspecific symptoms such as rash, myalgia, mood changes, altered mental status, libido changes, and peripheral edema. Signs consistent with adrenal insufficiency, including hypotension and weakness, were reported in 9.3% of cases. Overall, the clinical presentation was often nonspecific and insidious, which may delay diagnosis. However, the high prevalence of headache and fatigue in the context of recent ipilimumab therapy underscores the importance of prompt endocrine evaluation when these symptoms arise during the early treatment period.

### Laboratory findings

Laboratory data were available for the majority of included patients and demonstrated a characteristic pattern of anterior pituitary dysfunction (**Table 2**). Secondary adrenal insufficiency was the predominant endocrine abnormality. Among patients with available measurements, decreased serum cortisol levels were observed in 93.4%, and reduced adrenocorticotropic hormone (ACTH) levels were documented in 89.8%, indicating central hypocortisolism as the most frequent hormonal deficit. Thyrotropic axis involvement was also common. Low thyroid-stimulating hormone (TSH) levels were detected in 83.6% of evaluated patients, and reduced free thyroxine (FT4) was reported in 81.0%, consistent with central hypothyroidism. Gonadotropic dysfunction was similarly prevalent, with decreased follicle-stimulating hormone and/or luteinizing hormone levels in 84.2% of assessed cases. Prolactin abnormalities were less frequently reported but present in the majority of evaluated cases (85.7%), reflecting broader pituitary involvement. Hyponatremia was identified in 82.4% of patients with available sodium measurements, likely secondary to cortisol deficiency and impaired water balance. Overall, the laboratory profile was characterized by frequent multi-axis anterior pituitary impairment, with secondary adrenal insufficiency representing the most consistent and clinically significant abnormality. These findings underscore the importance of comprehensive hormonal assessment in patients presenting with suspected ipilimumab-induced hypophysitis.

**Table 2:** Clinical presentation and laboratory findings of 76 patients with ipilimumab-associated hypophysitis.

Parameter	Classification	Value
Clinical symptoms (75)^a^	Headache	51 (68.0%)
	Fatigue/malaise	48 (64.0%)
	Nausea/vomiting/anorexia	21 (28.0%)
	Hyponatremia	9 (12.0%)
	Adrenal insufficiency signs	7 (9.3%)
	Visual symptoms	8 (10.7%)
	Polyuria/polydipsia	4 (5.3%)
	Dizziness/vertigo	5 (6.7%)
	Gastrointestinal symptoms	4 (5.3%)
	Fever	3 (4.0%)
	Other symptoms (e.g., rash, myalgia, hot flushes/low libido, electrolyte abnormalities, altered mental status, uveitis, edema/cushingoid features, palpitations/tremors, arthralgia, etc.)	20 (26.7%)
Pituitary MRI (69)^a^	Pituitary enlargement/swelling	49 (71.0%)
	Stalk thickening/infundibulum involvement	15 (21.7%)
	Inhomogeneous/contrast enhancement	21 (30.4%)
	Other findings	6 (8.7%)
Laboratory tests		
Cortisol (61)^a^	Low (*Ref: 138–690 nmol/L*)	57 (93.4%)
ACTH (49)^a^	Low (*Ref: 7–63 pg/mL*)	44 (89.8%)
TSH (55)^a^	Low (*Ref: 0.4–4.0 mIU/L*)	46 (83.6%)
FT4 (42)^a^	Low (*Ref: 10–23 pmol/L*)	34 (81.0%)
PRL (7)^a^	Abnormal (*Ref: 4–25 ng/mL*)	6 (85.7%)
FSH/LH (38)	Low (*Ref: 1.5–12.4 IU/L*)	32 (84.2%)
Sodium (17)^a^	Low (*Ref: 135–145 mmol/L*), Hyponatremia	14 (82.4%)

^a^ Represents the number of patients with this parameter out of 76 patients;

^b^ Median (minimum, maximum).

ACTH, Adrenocorticotropic Hormone; TSH, Thyroid-Stimulating Hormone; FT4, Free Thyroxine; PRL, Prolactin; FSH/LH, Follicle-Stimulating Hormone/Luteinizing Hormone.

### Treatment and outcome

As shown in **Table 3**, glucocorticoid replacement constituted the cornerstone of management in patients with ipilimumab-induced hypophysitis. Hydrocortisone or cortisone acetate was the most commonly administered therapy (53.9%), followed by prednisone or prednisolone (35.5%). A smaller proportion of patients received methylprednisolone (10.5%) or dexamethasone (7.9%), particularly in cases presenting with more acute or severe symptoms. Hormone replacement targeting additional pituitary axes was frequently required. Levothyroxine supplementation was initiated in 39.5% of cases to manage central hypothyroidism, while testosterone replacement was provided in 11.8% of patients with hypogonadism. Fludrocortisone was prescribed in a minority of cases (3.9%), typically when mineralocorticoid support was indicated. Management of ipilimumab therapy varied according to clinical severity. Among cases reporting treatment modification, ipilimumab was discontinued in 66.7% and temporarily interrupted in 22.2%, whereas continuation or rechallenge was uncommon. Clinical improvement or symptom resolution was achieved in 82.9% of patients following hormone replacement therapy. However, long-term endocrine sequelae were common. Among 63 patients with available post-treatment recovery information, complete hormonal recovery was documented in 5 patients (7.9%), whereas persistent endocrine deficiency was reported in 44 patients (69.8%). In 14 patients (22.2%), clinical symptoms resolved but detailed hormonal recovery was not clearly reported. Mortality was reported in 3.9% of cases and was attributed to underlying cancer progression rather than hypophysitis itself. Rechallenge information was available in 57 patients. Ipilimumab rechallenge or resumption was documented in only 2 patients (3.5%), whereas 55 patients (96.5%) did not undergo rechallenge. Because post-rechallenge follow-up and endocrine outcomes were incompletely reported, the recurrence rate and safety of ipilimumab rechallenge could not be reliably estimated. Overall, while acute symptoms of ipilimumab-induced hypophysitis generally responded well to glucocorticoid and hormone replacement therapy, recovery of endogenous pituitary function was infrequent, indicating that permanent hypopituitarism is a common long-term outcome.

**Table 3:** Therapeutic strategies and clinical outcomes in 76 cases of ipilimumab-associated hypophysitis.

Parameter	Classification	Value
Treatment (76)^a^	Hydrocortisone/Cortisone acetate	41 (53.9%)
	Prednisone/Prednisolone	27 (35.5%)
	Methylprednisolone	8 (10.5%)
	Dexamethasone	6 (7.9%)
	Levothyroxine	30 (39.5%)
	Testosterone replacement	9 (11.8%)
	Fludrocortisone	3 (3.9%)
Ipilimumab management (18)^a^	Discontinued/stopped	12 (66.7%)
	Interrupted	4 (22.2%)
	Continued/restarted	2 (11.1%)
Outcome (75)^a^	Clinical improvement/resolution	63 (82.9%)
	Persistent endocrine dysfunction	32 (42.1%)
	Death (cancer progression related)	3 (3.9%)
Hormonal recovery (63)^a^	Axis recovered	5 (7.9%)
	Persistent endocrine deficiency	44 (69.8%)
	Resolved (clinical only)	14 (22.2%)
Ipilimumab rechallenge (57)^a^	Yes	2 (3.5%)
	No	55 (96.5%)
WHO-UMC causality category (76)^c^	Probable	61 (80.3%)
	Possible	15 (19.7%)

^a^ Represents the number of cases describing this parameter out of 76 patients.

^b^ Median (minimum, maximum).

^c^ According to the WHO–UMC causality framework, a case classified as “certain” demonstrates a well-defined temporal association with drug exposure, resolution following drug discontinuation, and recurrence upon rechallenge. A “probable” case presents with a plausible temporal relationship, lacks a more convincing alternative explanation, and shows clinical improvement after withdrawal without the need for rechallenge confirmation. A “possible” case also exhibits a reasonable time association; however, competing etiologies cannot be excluded, and the response to drug withdrawal may be inconclusive.

## Discussion

In this retrospective synthesis of 76 published cases, we comprehensively characterized the demographic features, clinical spectrum, endocrine abnormalities, radiologic findings, therapeutic strategies, and long-term outcomes of ipilimumab-induced hypophysitis. Our findings provide a consolidated overview of this distinctive immune-related endocrine toxicity and offer several clinically meaningful insights. A key observation is the early onset pattern of hypophysitis. The median time to presentation was 9 weeks, with more than half of cases occurring within the first 10 weeks of therapy. This relatively narrow window supports previous observations that CTLA-4–associated endocrinopathies most commonly develop during the induction phase of treatment, typically between the second and fourth infusion cycles (69). The consistency of this timing suggests a dose-independent immune priming mechanism rather than cumulative toxicity (5, 26). From a clinical perspective, this predictable onset period highlights the importance of structured biochemical monitoring and symptom surveillance during the early phases of ipilimumab therapy. The clinical manifestations were frequently nonspecific and potentially misleading. Headache and fatigue predominated, reflecting both pituitary enlargement and evolving secondary adrenal insufficiency. Because these symptoms overlap with cancer-related fatigue, chemotherapy effects, or dehydration, the diagnosis may be delayed if endocrine causes are not promptly considered. Importantly, some patients presented with hyponatremia or hypotension, indicating clinically significant adrenal dysfunction. Delayed recognition of central adrenal insufficiency can result in adrenal crisis, underscoring the need for immediate cortisol assessment in patients receiving CTLA-4 blockade who develop unexplained systemic symptoms (70).

Neuroimaging findings were characteristic but heterogeneous. The majority of patients demonstrated diffuse, symmetric pituitary enlargement with homogeneous enhancement, sometimes accompanied by infundibular thickening. This radiologic pattern differs from pituitary metastasis, which more commonly presents with nodular or asymmetric involvement. However, a proportion of patients exhibited normal magnetic resonance imaging, particularly when imaging was performed early or after partial inflammatory resolution. These findings reinforce that biochemical evaluation remains central to diagnosis and that normal imaging does not exclude immune-mediated hypophysitis. Endocrine evaluation revealed a consistent pattern of anterior pituitary dysfunction. Secondary adrenal insufficiency was the most prevalent abnormality, followed by central hypothyroidism and hypogonadotropic hypogonadism. This multi-axis involvement supports the concept that CTLA-4 blockade induces a diffuse inflammatory process within the adenohypophysis. Proposed mechanisms include ectopic CTLA-4 expression in pituitary endocrine cells and antibody-mediated complement activation leading to cellular injury (71). Unlike primary autoimmune hypophysitis, which often affects younger women and may involve the posterior pituitary, ipilimumab-induced hypophysitis predominantly affects the anterior lobe and typically spares vasopressin secretion (72). Nevertheless, rare cases of atypical hormonal dynamics, including transient hypercortisolism preceding adrenal failure, illustrate the evolving inflammatory process and emphasize the importance of serial hormone assessment rather than reliance on a single measurement (72). Management strategies were relatively consistent across reports. Glucocorticoid replacement was the cornerstone of therapy and resulted in rapid symptomatic improvement in most patients. High-dose corticosteroids were frequently administered during the acute phase, particularly in cases with significant mass effect or severe systemic manifestations. However, whether supraphysiologic immunosuppression influences long-term endocrine recovery remains uncertain, and the optimal intensity of glucocorticoid therapy continues to be debated. Current evidence does not clearly demonstrate that high-dose glucocorticoids improve pituitary recovery compared with physiologic replacement. Moreover, their potential impact on antitumor efficacy should be carefully considered. Given the inconsistent reporting of steroid dose, treatment duration, endocrine follow-up, and oncologic outcomes across the included cases, our data were insufficient to compare the effectiveness and safety of these management strategies. Notably, although acute symptoms improve, restoration of endogenous pituitary function is uncommon. Persistent hormonal deficiencies were observed in a substantial proportion of patients, indicating that CTLA-4–associated hypophysitis often results in irreversible pituitary damage. This pattern differs from certain other immune-related endocrinopathies, such as thyroiditis, in which partial recovery is more common.

Ipilimumab rechallenge or resumption was rarely reported, with only 2 of 57 patients undergoing rechallenge or resumption after stabilization. Because post-rechallenge outcomes were incompletely described, the recurrence risk and safety of rechallenge remain uncertain, and individualized decision-making with close endocrine monitoring is warranted. The decision to continue or discontinue ipilimumab varied and appeared to depend on toxicity severity and oncologic response. This individualized approach reflects the need to balance the survival benefits of CTLA-4 blockade against the risk of permanent endocrine morbidity. Interestingly, several studies have suggested a potential association between immune-related adverse events and enhanced antitumor efficacy, possibly reflecting more robust immune activation (73, 74). Although causality has not been established, this hypothesis highlights the complex interplay between therapeutic immune activation and autoimmunity. When compared with PD-1 or PD-L1 inhibitors, CTLA-4 blockade demonstrates a distinct endocrine toxicity profile. Hypophysitis is substantially more frequent with CTLA-4 inhibitors, whereas thyroid dysfunction predominates during PD-1-directed therapy. PD-1/PD-L1 inhibitor-associated hypophysitis is comparatively rare and often presents with normal pituitary MRI findings. In addition, it typically manifests as isolated ACTH deficiency rather than broad anterior pituitary involvement, contrasting with the multi-axis pituitary dysfunction and frequent pituitary enlargement observed in ipilimumab-induced hypophysitis (75). These differences likely reflect distinct immunobiological mechanisms, as CTLA-4 inhibition acts earlier in T-cell activation and may trigger broader immune amplification, potentially explaining the higher incidence of multisystem immune-related events, including pituitary inflammation (75, 76). Accordingly, early diagnosis, appropriate glucocorticoid replacement or corticosteroid therapy according to clinical severity, and prolonged endocrine surveillance are essential, given the variable course of ipilimumab-induced hypophysitis and the potential for irreversible pituitary dysfunction (77).

### Limitations of the study

Several limitations merit consideration. First, the retrospective case-based design introduced heterogeneity in diagnostic criteria, laboratory reference ranges, treatment regimens, and follow-up duration. Second, approximately one-third of patients received ipilimumab plus nivolumab, and combination immunotherapy may influence the onset, severity, and clinical phenotype of hypophysitis. However, incomplete case-level reporting precluded a reliable subgroup comparison between ipilimumab monotherapy and combination therapy. Third, incomplete endocrine reassessment and variability in reporting recovery may have underestimated partial hormonal recovery. Finally, publication bias toward symptomatic or severe cases is possible. Nonetheless, the relatively large pooled cohort enhances understanding of this rare but clinically significant complication.

## Conclusion

Collectively, our findings reinforce that ipilimumab-induced hypophysitis is a predictable early-onset toxicity characterized predominantly by secondary adrenal insufficiency and frequent pituitary enlargement. Although clinical symptoms typically respond to hormone replacement, long-term endocrine recovery is uncommon, necessitating prolonged surveillance and individualized management. As immune checkpoint inhibitors continue to expand across oncologic indications, improved awareness, standardized monitoring strategies, and multidisciplinary collaboration between oncologists and endocrinologists will be essential to optimize patient safety while preserving therapeutic benefit.

## Data Availability

The original contributions presented in the study are included in the article/**Supplementary Material**. Further inquiries can be directed to the corresponding author.
